# Commentary: Impact of a deletion of the full-length and short isoform of p75NTR on cholinergic innervation and the population of postmitotic doublecortin positive cells in the dentate gyrus

**DOI:** 10.3389/fnana.2016.00014

**Published:** 2016-02-19

**Authors:** Mohamed A. Sabry, Mona Fares, Ronnie Folkesson, Mariam Al-Ramadan, Jarrah Alabkal, Ghada Al-Kafaji, Moustapha Hassan

**Affiliations:** ^1^Department of Medical Biochemistry, College of Medicine and Medical Sciences, Arabian Gulf University Manama, Bahrain; ^2^Experimental Cancer Medicine, Laboratory Medicine, Karolinska Institute Stockholm, Sweden; ^3^Department of Neurobiology, Care Sciences and Society, Karolinska Institute Stockholm, Sweden; ^4^Biotechnology Program, College of Postgraduate Studies, Arabian Gulf University Manama, Bahrain; ^5^Department of Molecular Medicine/Al-Jawhara Centre for Molecular Medicine, Genetics and Inherited Disorders, College of Medicine and Medical Sciences, Arabian Gulf University Manama, Bahrain

**Keywords:** p75NTR, FL-p75NTR, s-p75NTR, RACE, western blotting, RT-PCR

We read with great interest the article by Poser et al. (2015). Full-length human/murine P75NTR (FL-p75NTR; ENST00000172229, ENSMUST00000000122, ENSRNOT00000007268) is encoded by six exons. Four cysteine-rich repeats span P75NTR extracellular domain and exhibit high-pro-neurotrophins-affinity and low-affinity to mature neurotrophins.

Brain P75NTR plays pivotal roles in neurogenesis. Embryonic neurogenesis derives from ventricular zone radial glia (Mochida et al., 2007). Adult neurogenesis is localized to stem cell niches in hippocampal dentate gyrus and the sub-ventricular zone (SVZ). P75NTR is detected in deep layers of developing neocortex (Bishop et al., 2002) and defines adult neurogenesis stem cells in SVZ (Young et al., 2007) and hippocampus (Catts et al., 2008). P75NTR regulates synaptic plasticity through its interactions with pro-BNDF to produce long-term depression (Woo and Lu, 2009). P75NTR Interacts with Nogo/Lingo-1 leading to growth cone collapse, neurite retraction, and spine density decrease (Meeker and Williams, 2015).

An alternative 62 kD P75NTR variant, named short isoform (s-p75NTR), claimed to arise by alternative splicing of P75NTR exon III, has been reported in the mouse and was claimed to also exist in rat and human species (von-Schack et al., 2001; Naumann et al., 2002). Exon III encodes cysteine-motifs 2–4 (Casaccia-Bonnefil et al., 1999). Its deletion would compromise P75NTR interaction with several molecules including Nogo (see above), sortilin and Trk. P75NTR-sortilin binding increases pro-neurotrophin affinity and promotes apoptosis while P75NTR-Trk interaction mediates pro-survival functions (Meeker and Williams, 2015).

To verify the existence of the presumed mouse s-p75NTR, we conducted Rapid Amplification of cDNA Ends (RACE) cloning on mouse P75NTR mRNA and ascertained many exon III-containing clones without any evidence of continuous exon II/exon IV sequence with exon III-skipping (Figure 1I). Also we conducted RT-PCR using oligos flanking exon III and validated our results by sequencing the identified PCR fragments (Figure 1II). Additionally, we used the same discontinuous, exon III-skipping primers which were used in the original reports as “specific” oligos to the presumed s-75NTR variant (von-Schack et al., 2001). These specific primers contain exons 2/4 boundary sequences. Also, this approach did not identify any short, exon III-excluding isoform in the mouse (Figure 1II).

**Figure 1:**
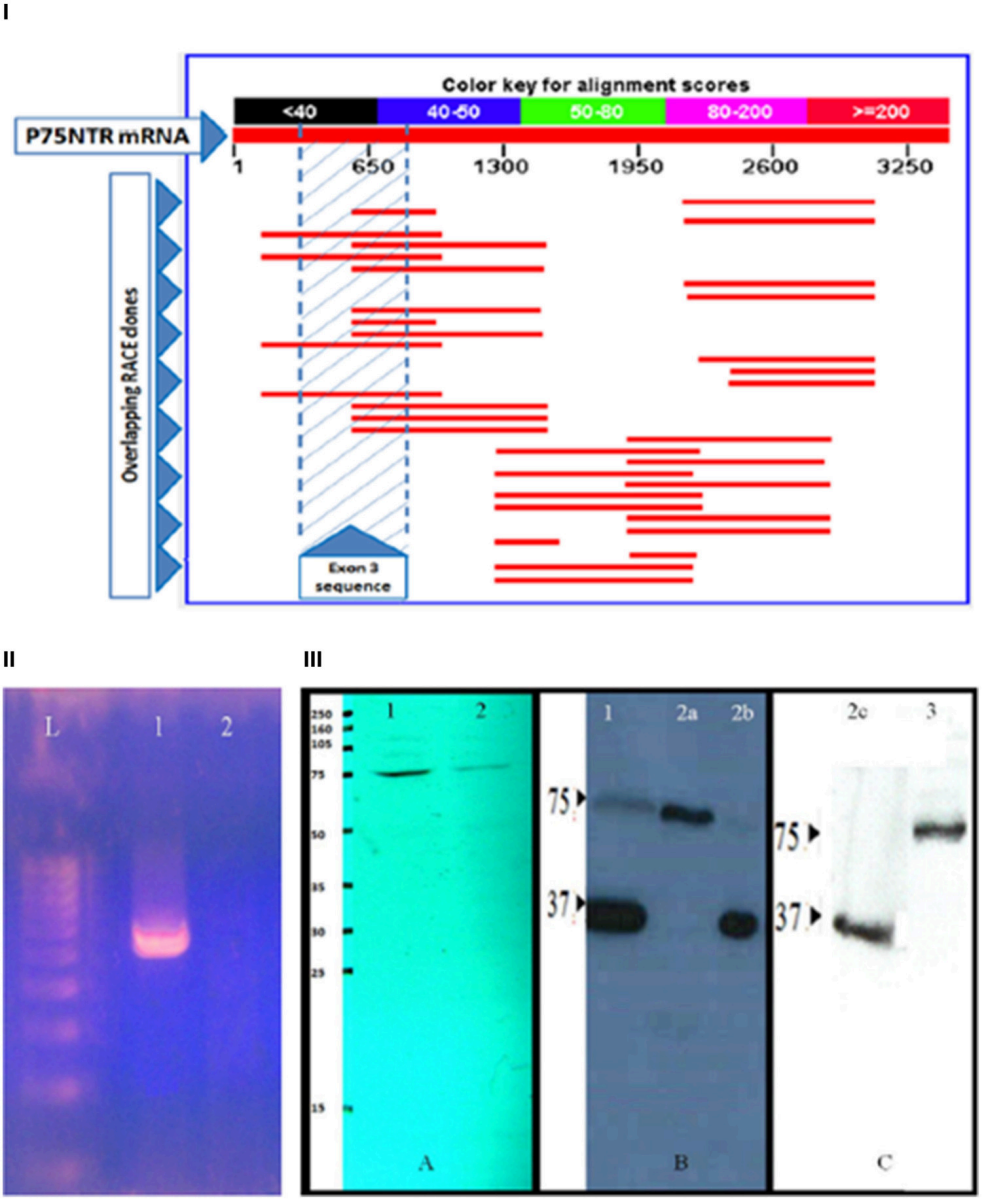
**Verifying P75NTR isoforms. (I)** RACE cloning to explore potential additional P75NTR variants: RACE cloning was conducted using a 225 bp template representing mouse brain P75NTR exon 4 nucleotides 781–1005. A large number of clones were screened, sequenced and BLASTed against mouse Refseq database (NCBI). Several exon III-containing clones (red lines in the shaded area) were ascertained while none of the screened clones showed exon III-skipping of the claimed s-p75NTR. **(II)** Exploring mouse brain cDNA for the presence of s-p75NTR: RT-PCR amplification was conducted using exon III-flanking primer pair 5′CCTGCCTGGACAGTGTTACG-3′/5′-GCCAAGATGGAGCAATAGACAG-3′ in lane (1) for FL-p75NTR amplification & exon III-skipping primer pairs 5′-TGCCTGGACAAGATCCCTGG-3′/5′-GGCCTGAGGCAGTCTGTGTG-3′ in panel (2) for possible amplification of the claimed s-p75NTR. RT-PCR was followed by electrophoresis on 2% agarose gel. In lane 1, a 550 bp band was amplified which, on sequenced, was found to represent FL-p75NTR (contains exon III). No amplification was detected for the claimed s-p75NTR in lane 2. L = 100 bp ladder, 1 and 2 = Adult mouse brain cDNA. **(III)** Exploring murine/human P75NTR protein variants. SDS-PAGE followed by WB of adult brain protein extracts from mouse (lane A-1), rat (lane A-2), human control (lane B-1), Alzheimer's disease (lanes B-2a, B2b, and C-2c), and CLL cell line K562 (lane C-3) using rabbit polyclonal antibody ANT-011 (Almone Labs) reactive to peptide CKQNKQGANSRPVNQT corresponding to residues 278–293 of human P75NTR (SwissProt accession P08138) with cross-reactivity to mouse and rat P75NTR. Only one band (75 kD), corresponding to FL-p75NTR is detected in protein extracts from mouse and rat. No 62 kD band (of the claimed s-p75NTR) was detected in either mouse, rat, adult human brain or human CLL cell line K562. Two protein bands (75 and 36 kD) are identified in protein extracts from human brain control/Alzheimer's disease corresponding to FL-p75NTR and the newly identified 36 kD human P75NTR variant.

Western blotting (WB) on mouse, rat, and human brain protein extracts using polyclonal antibodies specific to the intracellular region of P75NTR (Figure 1III) identified the 75 kD FL-p75NTR in the three species while a 62 kD band (corresponding to the claimed s-p75NTR) was not detected in any of the three species investigated. Apart from 75 kD FL-p75NTR, no other bands were detected in mouse and rat brains (Figure 1III). Of note is the fact that the report by (von-Schack et al., 2001) failed to show the protein band corresponding to the presumed 62 kD s-p75NTR in their WB. They rather showed a WB “smear” around 75 kD and argued that the 62 kD s-p75NTR band has been masked by the high level expression of FL-p75NTR! This is hardly convincing since the issue could have been easily resolved by simple technical improvements. It is noteworthy that our results, verifying only one murine FL-p75NTR band, are compatible with the documentations in major resources like Ensembl, NCBI, and UniProt database.

In addition to the 75 kD FL-p75NTR, our WB results on control and Alzheimer's disease human brains also detected an additional, previously unreported, human 36 kD isoform, (Figure 1III). The 36 kD P75NTR isoform does not seem to be a marker for Alzheimer's disease since we detected the two isoforms (75 and 36 kD), either simultaneously or alternatively, in individual human samples from control or Alzheimer's brains (Figure 1III). It is notable that three protein-coding P75NTR human transcripts (ENST00000172229, ENST00000504201, and ENST00000509200) are listed in ENSEMBL and UniProt. We are embarking on further analysis to determine the nature of our 36 kD isoform.

It is seriously alarming that the report by von-Schack et al. (2001) did not show any experimental work to support their claim for the presence of a 62 kD s-p75NTR. In addition to the lack of WB evidence, they did not even sequence their RT-PCR fragments to prove their claims. Such unsubstantiated reporting caused considerable confusion in the neuroscience community since other groups (Fujii and Kunugi, 2009; Poser et al., 2015) innocently reiterated such un-validated reports and, moreover, interpreted their important results based on such unfounded claims (Poser et al., 2015).

## Author contributions

All authors listed, have made substantial, direct and intellectual contribution to the work, and approved it for publication.

## Funding

This project is supported by grants 43/46AGU and by the Masters Program-Biotechnology-AGU.

### Conflict of interest statement

The authors declare that the research was conducted in the absence of any commercial or financial relationships that could be construed as a potential conflict of interest.
